# Role of Tibial Tuberosity Fracture/Fissure through the Maquet Hole in Stifle Osteoarthritis after Porous Tibial Tuberosity Advancement in Dogs at Mid-Term Follow-Up

**DOI:** 10.3390/vetsci7010001

**Published:** 2019-12-22

**Authors:** Alberto Maria Crovace, Francesco Staffieri, Donato Monopoli, Alejandro Artiles, Laura Fracassi, Antonio Crovace, Luca Lacitignola

**Affiliations:** 1 IRCCS “Saverio de Bellis”, Castellana Grotte, 70013 Bari, Italy; alberto.crovace@libero.it; 2 Dipartimento dell’Emergenza e dei Trapianti di Organi (DETO), Sezione di Cliniche Veterinarie e P.A, Università degli Studi di Bari “Aldo Moro”, s.p. per Casamassima Km 3. Valenzano, 70010 Bari, Italy; francesco.staffieri@uniba.it (F.S.); antonio.crovace@uniba.it (A.C.); 3 Instituto Tecnológico de Canarias, Santa Cruz de la Palma, 38009 Las Palmas, Spain; dmonopoli@itccanarias.org; 4 Dottorato di ricerca in “Trapianti di Tessuti ed Organi e Terapie Cellulari”, Dipartimento dell’Emergenza e dei Trapianti di Organi (DETO), Università degli Studi di Bari “Aldo Moro”, 70010 Bari, Italy; a.artiles@hvtarahales.es (A.A.); l.fracassi123@gmail.com (L.F.)

**Keywords:** tibial tuberosity advancement, complication, cranial cruciate ligament, dog

## Abstract

Tibial tuberosity advancement (TTA) is used to treat cranial cruciate ligament rupture of the stifle joint in dogs. Tibial tuberosity fracture/fissure is a complication of TTA that may have a favorable prognosis. The aim of this study was to detect how tibial tuberosity fracture/fissure through the Maquet hole worsens the progression of osteoarthritis (OA) in the stifle joint of dogs treated with porous TTA. Seventeen cases were included in the study, divided into two groups. The first group (*n* = 10) included subjects that had tibial tuberosity fracture/fissure through the Maquet, and the second group included subjects that had no complications (*n* = 7). Both groups showed significant progression compared to OA at 3 months after surgery. We observed that at T0, the control group showed a higher level of OA. For this reason, we normalized the OA scores, evaluating the percentage difference from T0 and T1. We verified that there were no statistically significant differences between the two groups. The results confirm that OA progression in subjects undergoing TTA was not significantly influenced by fracture/fissure of the tibial tuberosity through the Maquet hole. Therefore, fracture fissure through the Maquet hole should be considered as a common minor complication during TTA.

## 1. Introduction

Tibial tuberosity advancement (TTA) has been added to the set of surgical procedures used to treat cranial cruciate ligament (CCL) rupture of the stifle joint in dogs. Although the modification of the stifle joint geometry obtained with the TTA procedure has the aim of neutralizing cranial tibial subluxation, it does not restore the position of the tibia in relation to the femur, resulting in progression of osteoarthritis (OA) [1,2,3].

In this surgical area, different techniques have been described since the original Montavon procedure was reported [4]. Later, the modified Maquet technique was derived from human surgery and applied to dogs [5]. This technique uses a preplaced drill hole (Maquet hole) at the proposed termination site of the osteotomy to prevent fissure or propagation of the osteotomy past this predetermined location [5,6]. Nevertheless, the risk of fracture of the distal tibial tuberosity, or even the tibia, from propagation of the osteotomy was described in 20% of procedures [5]. 

In previous studies, post-TTA complications included tibial fracture, rupture of the implant, meniscal lesions, medial patellar luxation, complete tear of incompletely torn CCL, and infection [7,8,9,10,11,12,13,14,15,16]. Tibial tuberosity fracture occurred intraoperatively and was described as an incidental finding on follow-up [7,8,9,17,18]. Calvo et al. [17] stated that tibial tuberosity fracture is a complication of tibial tuberosity advancement that may have a favorable prognosis [5], although it can result in significant morbidity, and in some cases revision surgery may be required [17].

Porous TTA was recently described with the use of the Maquet technique, in which a porous 3D biomimetic titanium cage was inserted to provide the tibial tuberosity advancement [19]. The aim of this study was to detect how tibial tuberosity fracture/fissure through the Maquet hole worsens the progression of osteoarthritis (OA) in the stifle joint of dogs treated with porous TTA.

## 2. Materials and Methods 

### 2.1. Population

**Figure 1:**
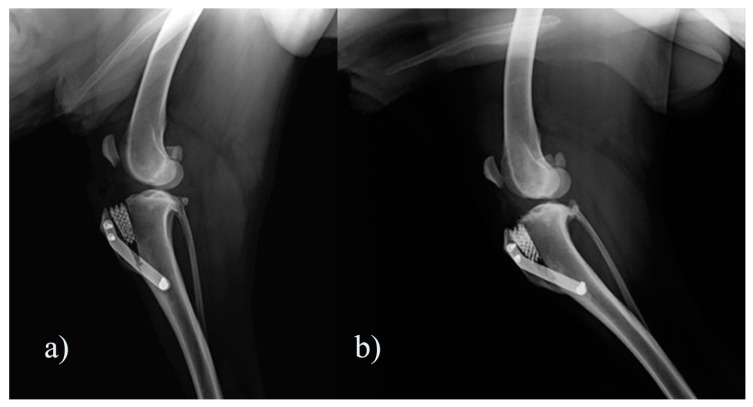
Representative images of mediolateral X-ray view of a case included in FX group (**a**) postoperatively and (**b**) 3 months after surgery.

Seventy-five cases of dogs subjected to porous TTA according to the technique described in a previous study were retrospectively examined [19]. Inclusion criteria consisted of dogs that had postoperative X-ray examinations (T0) and were 3 months post surgery (T1), showing no implant failure and no complications other than tibial tuberosity fracture/fissure through the Maquet hole. Cases of surgical revision of previous repair surgeries of CCL rupture, other reported complications, and X-rays not available 3 months after surgery were excluded. The subjects were divided into two groups: the first group (Fx group) included subjects that had tibial tuberosity fracture/fissure through the Maquet (Figure 1), and the second group included subjects with no complications (No Fx group).

### 2.2. Evaluation of Osteoarthritis

Immediate postoperative X-rays and 3 month postoperative examinations were then evaluated. Five independent observers with different experience in evaluating the degree of osteoarthritis evaluated radiograms in mediolateral and cranial caudal views. The staging protocol for osteoarthritis of the knee was evaluated by applying a method modified from the one suggested by Wessely in 2017 [20], eliminating from the analysis the anatomical points relating to the tibial tuberosity, as they were considered not assessable in the course of TTA.

In this protocol, the knee joint was divided into 13 anatomical points of interest: patellar apex, patellar base, proximal trochlear tuberosity, distal trochlear tuberosity, femoral condyle, plateau tibial caudal aspect, plateau appearance, central tibial, femoral, popliteal surface, sesamoid bones, lateral femoral/tibial condyles, medial femoral/tibial condyles, intercondylar notch, patella. For each point, a score from 1 to 4 was assigned depending on the severity of typical OA findings: 1, normal radiographic appearance, absence of sclerosis or osteophytes; 2, slight osteophytosis and/or slight sclerosis; 3, moderate osteophytosis and moderate sclerosis; 4, marked osteophytes and severe sclerosis. 

### 2.3. Statistical Analysis

The data obtained were analyzed with MedCalc 14 software (MedCalc Software, Ostend, Belgium). The scores were then analyzed to evaluate the presence of statistically different variations between observers. The Kruskal–Wallis test was performed to evaluate differences between the scores and the related differences, obtained at T0 and T1 comparing Fx and control groups. Furthermore, we calculated and compared the percent of increment of OA score between follow-ups. Significance level was detected at *p* < 0.05.

## 3. Results

### 3.1. Population

Sixteen cases met the inclusion criteria, of which one subject had bilateral rupture, for a total of 17 stifle joints examined. Fifty-eight cases did not reach the 3 month postoperative follow-up, did not return for radiographic recheck, or had unavailable complete clinical exams and X-rays. The mean weight was 29.6 kg (±12.0). This population included seven female and nine male dogs consisting of three mixed, two Breton, two Dogo, two Segugio Maremmano, one Central Asia shepherd, one golden retriever; one Labrador retriever, one Rottweiler, one Samoyed, one beagle, one Shar-Pei, and one Irish setter.

Seven stifle joints were included in the control group (No Fx group) and 10 in the group with fracture/fissure of the Maquet hole (Fx group). No fracture in this group was fixed or reoperated, and they were conservatively managed.

The incidence of fracture/fissure through the Maquet hole in the cases that matched the inclusion criteria was 58.8% (10 to 17). 

### 3.2. Evaluation of Osteoarthritis

**Figure 2:**
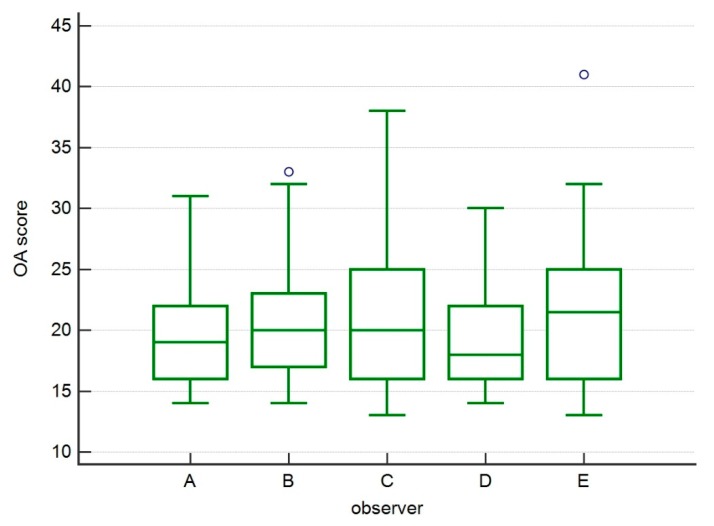
Box and whisker graph of OA scores by observers enrolled for the study. No statistically significant differences were detected, showing a homogeneous evaluation among different observers.

The statistical analysis of variability among the observers showed no statistically significant changes, showing a homogeneous evaluation among the different observers (Figure 2).

**Figure 3:**
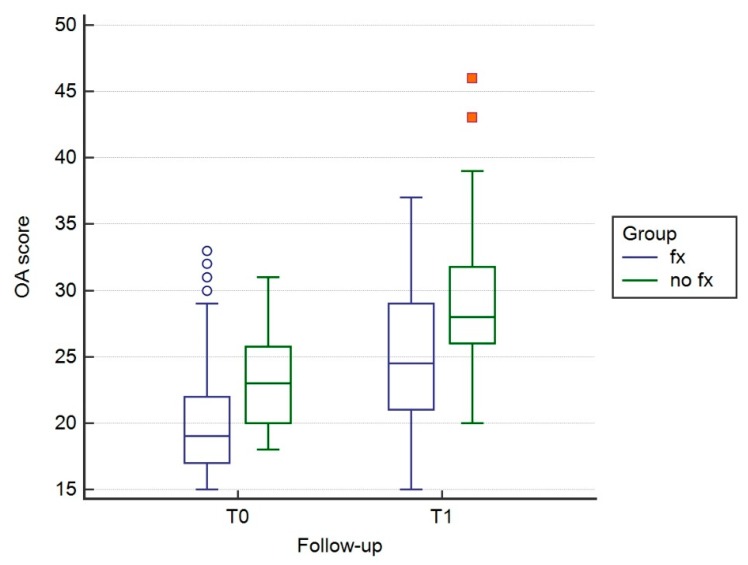
Box and whisker graph of OA scores at T0 and T1 for Fx and No Fx groups. A significant increase of OA score was observed in both groups at respective follow-up (*p* < 0.01).

**Figure 4:**
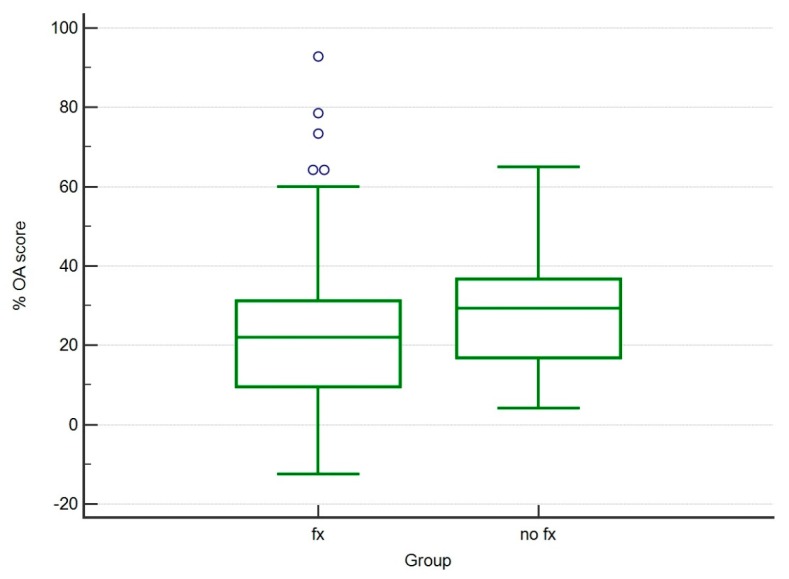
Box and whisker graph of percentage increase of OA score between Fx and control groups (*p* > 0.05).

The scores obtained by the various observers showed postoperatively higher OA score in the control group (no Fx) compared to the Fx group (*p* < 0.005) (Table 1.). Three months after surgery (T1), a significant increase of the OA score was observed in both groups (*p* < 0.005) (Figure 3). The % increment of OA score was not statistically different between observed groups (*p* > 0.05) (Table 2; Figure 4).

## 4. Discussion

In this retrospective study, we evaluated the progression of osteoarthritis in subjects undergoing porous TTA that developed fracture or fissure through the Maquet hole by comparing them with a control group that did not develop complications.

To assess the OA stage, we used a scoring system already validated and described previously [3,20] but modified for this specific study. In particular, we removed the anatomical points relative to the tibial tuberosity from the evaluation, because during TTA it is affected by the osteotomy, and during healing it presents radiographic changes as a function of bone repair of the osteotomy line and integration of the titanium cage. 

Furthermore, in this study, different observers with different clinical experience evaluated the radiographic images blindly in order to score the OA more objectively. The results showed no significant differences among the observers, thus showing that this was a homogeneous evaluation and the OA evaluation method was simple and objective. 

We observed that at T0 the control group showed a higher level of OA. For this reason, we normalized the OA scores, evaluating the percentage difference from T0 and T1. In this way, we verified that there were no statistically significant differences between the two groups. This confirms that OA progression in subjects undergoing TTA was not significantly influenced by the fracture/fissure of the tibial tuberosity through the Maquet hole. Studying the cause of OA progression in the stifle joint in the course of TTA was not the objective of this study, although other studies have considered the potential risk factors for the development of OA in an affected stifle joint [3,21,22].

Interestingly, both groups showed significant progression compared to OA at 3 months after surgery, in accordance with the bibliographic data. In fact, it was found that 55% of the treated stifle joints presented progressive OA within 4–16 months of TTA intervention [3,7,21,22]. 

One previous hypothesis suggested that the progression of new bone formation was higher in dogs with severe cartilage lesions at the time of surgery and that meniscal lesions contributed to faster progression of OA [22]. Moreover, it has been reported that extensive arthrotomy and removal of CCL remnants may predispose subjects to increased progression of OA [21,23]. However, severity of radiographic OA does not correlate well with clinical function [23].

The present study did not evaluate the possible etiology of the development of tibial tuberosity fracture or fissure through the Maquet hole in the course of porous TTA. Many studies suggested that reduced thickness of the osteotomized tibial tuberosity, incorrect plaque positioning, reduced contact of the osteotomy, wide angle of the preoperative patellar ligament, and iatrogenic region wounds during surgical dissection contribute to the development of this complication [8,17,18]. Lefebvre et al. [24] stated that intraoperative fissures occurred more frequently than intraoperative fractures and were located most commonly at the distal aspect of the osteotomy line. They also considered the angle of opening of the osteotomy line and the thickness of the cortical hinge as the main factors increasing the risk of perioperative tibial damage during Maquet modified technique (MMT) in dogs [24]. 

The data of the present study show that the incidence of tibial tuberosity fractures during porous TTA was 13.3%. The reported incidence ranged from 1–4% [7,8,25] to 20% [5]. In Lefebvre’s study [24], intraoperative fissures were detected in 37% of MMT cases, but only 9.4% subsequently led to postoperative tibial fracture. Based on published data reporting complication rates, an acceptable failure rate should be set at 15% and an unacceptable failure rate at 25% during the initial learning curve [17,26]. This was the most considerable complication as accidental identification of fractures during follow-up examinations. 

## 5. Conclusions

In our study, all fractures or fissures were conservatively managed. In our view, the lack of significant differences of OA scores in comparing the control group with no fixed fractured tibial tuberosity cases confirms that this complication does not significantly affect the progression of OA; therefore, fracture fissure through the Maquet hole should be considered common, with a minor impact on dogs.

## Figures and Tables

**Table 1:** Mean and SD of osteoarthritis (OA) score assigned for No Fx group (control) and Fx group at respective follow-up.

Group	Follow-Up	Mean	SD
**No Fx**	T0	19.3	3.2751
	T1	24.9	5.6253
**Fx**	T0	17	3.6978
	T1	21.1	4.8414

**Table 2:** Mean of % increment of OA score among observers ± SD.

Group	% OA Score Increment	SD
**No Fx**	29.20	15.02
**Fx**	25.31	22.89
